# Insights in the anode chamber influences on cathodic bioelectromethanogenesis – systematic comparison of anode materials and anolytes

**DOI:** 10.1002/elsc.201900126

**Published:** 2019-09-30

**Authors:** Franziska Enzmann, Markus Stöckl, Denise Gronemeier, Dirk Holtmann

**Affiliations:** ^1^ Evonik Industries Technology and Infrastructure Hanau Germany; ^2^ DECHEMA Research Institute, Electrochemistry Frankfurt am Main Germany; ^3^ DECHEMA Research Institute, Industrial Biotechnology Frankfurt am Main Germany; ^4^ Institute of Bioprocess Engineering and Pharmaceutical Technology University of Applied Sciences MIttelhessen Giessen Germany

**Keywords:** bioelectromethanogenesis, bioelectrosynthesis, counter electrode, electrolyte influence, linear sweep voltammetry

## Abstract

Cathode and catholyte are usually optimized to improve microbial electrosynthesis process, whereas the anodic counter reaction was not systematically investigated and optimized for these applications yet. Nevertheless, the anolyte and especially the anode material can limit the cathodic bioelectrochemical process. This paper compares for the first time the performance of different anode materials as counter electrodes for a cathodic bioelectrochemical process, the bioelectromethanogenesis. It was observed that depending on the anode material the cathodic methane production varies from 0.96 µmol/d with a carbon fabric anode to 25.44 µmol/d with a carbon felt anode of the same geometrical surface area. The used anolyte also affected the methane production rate at the cathode. Especially, the pH of the anolyte showed an impact on the system; an anolyte with pH 5 produced up to 2.0 times more methane compared to one with pH 8.5. The proton availability is discussed as one reason for this effect. Although some of the measured effects cannot be explained completely so far this study advises researchers to strongly consider the anode impact during process development and optimization of a cathodic bioelectrochemical synthesis process.

**Abbreviations**
DSA: dimensional stable anode
LSV: linear sweep voltammetry
MES: microbial electrosynthesis
MFC: microbial fuel cell

## 1 INTRODUCTION

Microbial electrosynthesis (MES) allows the production of valuable fuels and chemicals from electrical energy and carbon dioxide 1, 2, 3, 4. Examples for organic molecules which can be produced are methane, acetate, humulene, carboxylic acids, and alcohols 2, 4, 5, 6. The technology usually combines the metabolic activity of electroactive micro‐organisms as catalysts at a cathode with an abiotic electrochemical reaction at an anode, for example water splitting 5. In many cases, the used bioelectrochemical systems consist of two chambers, the cathode and the anode chamber, where the two reactions take place spatially separated from each other by an ion exchange membrane. This can be useful either to protect the micro‐organisms from toxic compounds formed at the anode (like e.g., oxygen) or to gain higher purities of the product 7, 8, 9. The systems used for bioelectrotechnology in general are less sophisticated than electrochemical systems in accordance to critical design parameters such as system resistance or used materials; often, electrodes in MES are carbon based due to the lower price compared to high costs of more efficient precious metal electrodes 1, 10.

Naturally, the cathode as working electrode often lies within the focus of research and optimization in MES technology. As an example, different carbon based electrodes with various structures were tested, carbon electrodes were combined to assemblies such as carbon sticks wrapped with carbon paper, or assemblies with metal wires 11. To further improve the performance, carbon materials were coated with metal particles or polymers 12. It was reported that chitosan treatment of a graphite cathode improved the acetate production by means of 7.7 12. Not only carbon materials, but also non‐precious metals were tested, however, the use of carbon materials seemed to allow higher production rates in many cases 13.

During working electrode optimization in another bioelectrochemical process, the microbial fuel cell (MFC), it turned out that the counter electrode (in case of an MFC the cathode) can be limiting for the desired process, although no biological reaction takes place at the counter electrode surface 14, 15. Comprehensive studies were carried out in MFCs showing that type and size of the counter electrode limit the current production. Oh et al. reported an improvement of current production in MFCs when coating the counter electrode with platinum 14. It was found that an enlarged counter electrode surface area improved the current production, but not in a proportional way 14. It was suggested that the counter electrode contributed to a large portion of the system resistance, limiting the electrochemical performance 16. Other conditions at the counter electrode, such as dissolved oxygen concentration or ferricyanide addition, also influence the MFC performance 14, 17.

In MES, the anode is the counter electrode, and to our knowledge, it has not systematically been studied so far about how the process can be optimized by alteration of the conditions at the anode. In this publication, we want to reveal whether and why different anodes materials influence the desired process at the cathode. Mainly, carbon based anode materials commonly used in bioelectrochemistry were chosen and compared. As an example process for MES, the bioelectrochemical production of methane by the electroactive methanogen *Methanococcus maripaludis* was chosen, which was already described in literature 5, 18, 19. Not only different anode materials, but also different anolytes are investigated and compared. Apart from that, we support the findings by electrochemical electrode characterization using linear sweep voltammetry (LSV), which shall give a better understanding of the influences the anode chamber has on the process of bioelectromethanogensis. This kind of systematic comparison of different counter electrode conditions was not shown before for cathodic processes.

**PRACTICAL APPLICATION**

The optimization of bioelectrochemical systems is a crucial step towards industrial applicability. The investigations shown in this paper suggest a systematic optimization of the counter chamber. This system part has not been studied before in a comparative manner. Researchers can transfer the results shown here to other bioelectrochemical systems to improve the process performance. For a future industrial application, counter electrode optimization is crucial to gain a sustainable and feasible process. Most probably it is easier to optimize the overall process by improving the abiotic electrode reaction than by improving the biotic reaction.

## 2 MATERIALS AND METHODS

### 2.1 H‐cell setup

The used H‐cells (Fischer Labortechnik, Frankfurt am Main, Germany) consisted of two 100 mL glass bottles connected via a glass bridge. To create a two‐chamber system, a membrane (Nafion117, DuPont, Wilmington, USA, 4.9*10^−4^ m²) was inserted in the bridge; Nafion is used as a standard in bioelectrochemistry due to the fact that it can be autoclaved 20. Including the side ports, the absolute volume of each chamber added up to 142 mL. As cathode, a graphite rod was used (0.5 cm diameter, 7.5 cm long; Metallpulver24, Sankt Augustin, Germany). Different materials were used as anodes (see Section 2.2). if not stated otherwise, a graphite rod was also used as anode. The electrodes were placed into each chamber and contacted with a titanium wire (0.5 mm diameter, Goodfellow, Bad Nauheim, Germany; 2 mm diameter in case of dimensionally stable anodes (DSA, De Nora, Milan, Italy)) The wires were pierced through a butylseptum (Glasgerätebau Ochs, Bovenden, Germany, septum for GL45) closing the main opening of each H‐cell chamber. The contacting titanium wire was not submerged in the electrolyte. The cathode chamber was equipped with a Luggin capillary (Fischer Labortechnik, Frankfurt am Main, Germany) filled with 0.5 M Na_2_SO_4_ holding an Ag/AgCl reference (Ag/AgCl electrode; +199 mV vs. SHE, SE 21, Sensortechnik Meinsberg, Xylem Analytics, Germany). Further septa (Glasgerätebau Ochs, Bovenden, Germany) closing the side arms of each chamber allowed gassing and sampling of headspace gas (gas inlet: (0.6*80 mm needle, gassing rate 0.5 mL/min N_2_/CO_2_ (80/20); gas outlet: 0.6*30 mm needle). A further cannula was inserted into the anode chamber for air exchange between anode chamber and environment and avoidance of overpressure by the production of oxygen at the anode. The three electrodes were connected to a potentiostat (Multi Master 2.1 potentiostat, Material Mates, Milano, Italy) with stainless steel alligator clips, setting the potential of −900 mV vs. Ag/AgCl between the cathode and the reference electrode. The current was monitored constantly. The terminal voltage E_cell_ was measured daily (OWON multimeter B35, Fujian Lilliput Optoelectronics Technology Co, Zhangzhou, China). The anode potential (E_an_) was calculated as E_an_ = ‐ (E_cell_ ‐ E_cath_) with the set cathode working potential E_cath_ of –900 mV vs. Ag/AgCl. The H‐cells were placed in an incubator hood, at a temperature of 35°C was set. The cathode chamber was filled with 100 mL of MES medium (0.35 g/L KCl, 4 g/L MgCl_2_·6H_2_O, 3.45 g/L MgSO_4_ ·7H_2_O, 0.25 g/L NH_4_Cl, 0.14 g/L CaCl_2_·2H_2_O, 0.14 g/L K_2_HPO_4_, 0.002 g/L Fe(NH_4_)_2_SO_4_, 18 g/L NaCl, 10 ml/L trace element solution DSMZ M141 and 10 ml/l vitamin solutions DSMZ M141, 5 g/L NaHCO_3_; all chemicals used are of analytical grade). The MES medium used was an alteration of the standard methanogenium medium M141 given by the DSMZ. If not stated otherwise, the anode chamber was filled with 100 mL of 100 mM phosphate buffer (pH 6.9; 5.62 g/L KH_2_PO_4_, 9.28 g/L Na_2_HPO_4_) to increase the conductivity.

### 2.2 Anode materials

Five different anode materials were tested: graphite rod, activated carbon felt, carbon fabric, DSA, and carbon laying. All anodes were contacted with titanium wire (as the cathodes), whereas the connecting titanium was not submerged in the electrolyte. The electrical connection led to different contact resistances among the materials due to their material properties. Details are given in Table 1. For all materials except DSA, the basic material was carbon, scanning microscopy images are given in Supporting Information. DSA is a titanium‐mesh with Ir‐MMO (mixed metal oxides) coating. In contrast to the carbon based electrodes, DSA showed a grid‐like structure. The carbon based materials offered a similar, but not exactly equal geometrical surface area, so current densities and specific methane production rates were also calculated based on the geometrical surface area of the anode to still allow comparison of the materials. The projected surface area of DSA was similar to the geometrical surface area of the carbon based materials, but due to the grid‐like structure, the geometrical surface area was much smaller.

**Table 1:** Properties of different anode materials for biotic experiments in H‐cells

Anode type	Graphite rod	Carbon felt (SIGRA THERM® GFA5)	DSA	Carbon laying	Carbon fabric ACC5092‐15
Geometrical surface area	0.00118 m²	0.00100 m²	0.0005 m²	0.00140 m²	0.00140 m²
Specific surface area (measured via physisorption)	25.23 m²/g	39.97 m²/g	n.a.^a^	0.89 m²/g	1635.29 m²/g
Specific resistance	0.05 mΩm	1.41 mΩm	5.70 mΩm	1.63 mΩm	13.98 mΩm
Supplier	Metallpulver24, Sankt Augustin, Germany	SGL Carbon, Wiesbaden Germany	De Nora, Milan, Italy	HP textiles, Schape, Germany	Kynol, Hamburg, Germany
Used mass	3.15 g	0.27 g	n.a.	0.75 g	0.27 g
Total surface area	79.43 m²	10.71 m²	n.a.	0.66 m²	446.60 m²
Contact resistance	0.7 Ω	3.1 Ω	0.5 Ω	4.4 Ω	109 Ω
Density	1595.7 kg/m³	96.8 kg/m³	n.a.	383 kg/m³	283.3 kg/m³

a The specific surface area of DSA could not be detected using physisorption since it is too low. The geometrical surface calculated via the free cross section: 25 % of the projected surface is 0.0005 m^2^

### 2.3 Anolytes

Five anolytes were tested addressing the anode chamber pH. First, 100 mM phosphate buffer pH 6.9 was used as anolyte (5.62 g/L KH_2_PO_4_, 9.28 g/L Na_2_HPO_4_). In the second and third, 100 mM phosphate buffer with a pH of 5 (0.16 g/L K_2_HPO_4_, 15.46 g/L NaH_2_PO_4_·2H_2_O) or 8.5 (17.3 g/L K_2_HPO_4_, 0.15 g/L NaH_2_PO_4_·2H_2_O) was used, respectively. In the fourth experiment, 0.1 M HCl solution was used as anolyte, and in the fifth, 0.1 M NaOH solution.

To enhance the anolytes conductivity, an experiment was done in which the phosphate buffer was replaced by 100 mL MES medium.

### 2.4 LSV

Abiotic characterization of the electrode materials was performed to demonstrate the different electrochemical behavior of the different materials and the influences of the pH on the electrochemical performance. LSV was chosen as method for evaluation. The same anode materials as in Section 2.2 were used, but the geometrical surface areas were altered. Carbon fabric (geometrical surface of 0.0002 m²), carbon felt (geometrical surface of 0.0002 m²), and carbon laying (geometrical surface of 0.0008 m²) were connected to a platinum wire (diameter 0.5 mm) to decrease the contact resistance for this experiment. The graphite rod was wrapped with PTFE tape in order to achieve a geometrical surface area of 0.00019 m² and electrically connected with a titanium wire (diameter 0.5 mm). The DSA electrode was used as delivered by the manufacturer (geometrical surface area: 0.0005 m², welded to a titanium wire, diameter 2 mm). The surface area of the electrodes used for the abiotic characterization was smaller than for the biotic experiments in H‐cells, since larger electrode areas would lead to current overloads when performing the LSV. Since the results are given in current densities based on the geometrical surface areas, conclusions may be transferred to larger electrodes. Images illustrating electrode materials and electrical connection are presented in the Supporting Information. The experiments were carried out in a 100 mL Schott flask (one‐chamber system, in contrast to the biotic chronoamperometric measurements) equipped with a lid with GL14 ports. The potential of the anode was controlled with an Ag/AgCl/KCl_sat_ electrode (+199 mV vs. SHE, SE 21, Sensortechnik Meinsberg, Xylem Analytics, Germany), inserted via a Luggin capillary filled with KCl_sat_. A platinum mesh (geometrical surface of 0.0012 m²) served as cathode during LSV experiments. The electrodes were each inserted through a respective GL14 port. An image illustrating the electrode positioning can be found in the Supporting Information. A 100 mM phosphate buffer was used as electrolyte at three different pH values, set by the ratio of hydrogen‐phosphate to dihydrogen‐phosphate (see Section 2.3).

Linear sweep experiments were carried out with a Gamry Reference 600 potentiostat (Gamry Instruments, Warminster, USA). LSV were started at 0 V vs. Ag/AgCl and driven to 2.5 V vs. Ag/AgCl with a scan rate of 100 mV/s and a step size of 2 mV. The resistance was uncompensated. The experiments were carried out at controlled room temperature of 20°C.

### 2.5 Biotic experiments

All experiments in H‐cells were conducted in two independent biological duplicates and one abiotic control. All chronoamperometric H‐cell experiments were operated at −900 mV vs. Ag/AgCl and 35°C (close to the temperature optimum of the used organism 21) for 80 h.

As electroactive organism, *Methanococcus maripaludis* S2 (DSM No.: 14266, DSMZ, Braunschweig, Germany) was used for the biotic experiments. The precultures for the inoculation were cultivated in 1 l septum flasks with 300 mL of M141 medium and 2 bar H_2_/CO_2_ 80/20 v/v gas atmosphere to an optical density of approximately 1 (late exponential phase) at 180 rpm and 37°C. The cathode chamber of the H cell was inoculated after sparging with N_2_/CO_2_ for half an hour to an OD of 0.1. During the experiments, the cathode chamber was continuously gassed with 5 mL/min N_2_/CO_2_ 80/20 v/v. This lead to an equilibrium of bicarbonate and CO_2_ and a pH of 7.2.

### 2.6 Analytics

Gas samples were taken from the H‐cells twice a day and analyzed via GC (Agilent technologies 490 Micro GC, Agilent, Santa Clara, USA (with external 2‐point‐calibration)). For analysis of the off gas samples, an injector temperature of 100°C and a column temperature of 60°C were set. Samples were injected to three columns: Channel 1: PoraPLOT U pre‐column and Molsieve 5A main column with argon as carrier gas; Channel 2 PoraPLOT U pre‐column and Molsieve 5A main column with helium as carrier gas; Channel 3 PoraPLOT U as pre column and main column with helium as carrier gas. A thermal conductivity detector was used. Hydrogen was detected on channel 1, oxygen and nitrogen on channel 2 and methane and carbon dioxide on channel 3. The sampling time was set to 30 s, the total runtime to 3 min. From the percentage of methane and hydrogen in the off‐gas stream, the production rate was determined using the gas flux and the molar standard volume. The mean values given in the results section were calculated using the mean values from 24 h after inoculation to the end of the experiment to exclude effects of initial electrode polarization and microbial lag phase during the start‐up phase and to avoid the measurement of residual gas form the pre‐culture. To calculate the Coulombic efficiency, Equation (1) was used with r_e,l_ as electron transfer rate from the electrode given by the current and r_e,m_ as electron transfer rate to the metabolite given by the methane production.

(1) $$\eta_{C,MES}=\frac{8*r_{e,m}}{r_{e,\;I}}\;$$

The Coulombic efficiency was calculated from the mean current and the mean methane production.

After the experiments with different anolytes, pH (VoltcraftPH100ATC; Voltcraft, Hirschau, Germany) and conductivity (HI99301 conductivity meter, Hanna instruments, Vöhringen, Germany) were measured in the anode and cathode chamber. After all experiments, the optical density at the end of the chronoamperometric experiment was measured (WPA Biowave CO8000 Cell Density Meter, 600 nm, Biochrom, Cambridge, England).

## 3 RESULTS AND DISCUSSION

### 3.1 Effect of anode material

**Figure 1:**
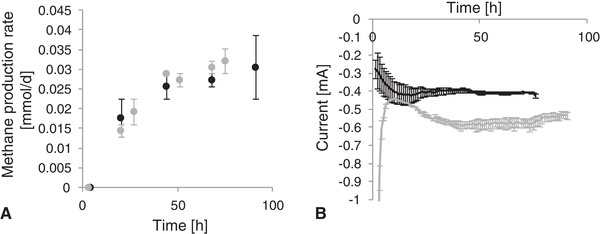
Methane production rates and current consumption. (A) Methane production at the cathode side in bioelectromethanogenesis; Black dots: biotic methane production rate using carbon felt as anode; grey dots: biotic methane production rate using graphite rod as anode. (B) Current consumption during bioelectromethanogenesis using different anodes; solid black line: using carbon felt as anode; solid grey line: using graphite rod as anode

Five different electrodes were tested as anodes for the bioelectromethanogenesis. Exemplary, the current consumption and the methane production rate for the graphite rod and the carbon felt anode are shown in Figure 1 and Table 2. The methane production rate increased rapidly and remained relatively stable after 45 h, corresponding to a stable current. Previous studies showed that the process of bioelectromethanogenesis can be operated with stable methane production rates over longer periods of time 22, therefore the results obtained here within 80 h are considered as representative. The current uptake was larger with the graphite rod anode than with the carbon felt (Figure 1B), although the methane production rates in both experiments were similar. Using the graphite rod as anode, a high current was observed in the beginning of the experiment, which decreased rapidly before the current increased again due to microbial current uptake. The initial current (first 10 h of the experiment) was excluded when calculating the mean current and mean efficiency, since it was assumed that the initial release of electrons was not connected to microbial methane production but polarization of the electrode surfaces. The first measured value of the methane production and the hydrogen production was also excluded for the calculation of the mean production rate since it might result from the gas phase from the preculture introduced to the H‐cell during inoculation. Randomized, samples were measured with HPLC, but in no case soluble organics such as acetate, formate or lactate were detected.

**Table 2:** Performance of biotic H‐cells using different anodes

Anode type	Graphite rod	Carbon felt	DSA	Carbon laying	Carbon fabric
Current density [mA/m²]	−474 ± 19	−250 ± 4	−960 ± 31	−400 ± 87	−307 ± 20
Anode potential [V]	0.885	0.834	0.997	0.949	0.694
CH_4_ production rate [µmol/d]	25.31 ± 4.42	25.44 ± 1.88	14.46 ± 2.89	15.54 ± 3.75	0.96 ± 0.32
CH_4_ production rate [mmol/d m^2^_Anode_]	21.45 ± 2.46	25.44 ± 1.88	28.92 ± 5.78	11.10 ± 2.68	0.69 ± 0.23
H_2_ production rate [µmol/d]	4.42 ± 1.33	11.25 ± 0.54	14.79 ± 7.81	5.89 ± 4.82	48.85 ± 14.79
Coulombic efficiency to CH_4_ [%]	40.1	56.1	26.9	24.8	2
Coulombic efficiency to H_2_ [%]	1.8	6.2	6.9	2.4	25.3

The highest absolute methane production rate was observed for carbon felt anodes (25.44 ± 1.88 µmol/d, equals 21.56 mmol/d m² based on the geometrical cathode surface area and 15.63 mmol/d m² based on the geometrical anode surface area), followed by the use of graphite rod anodes (25.31 ± 4.42 µmol/d, equals 21.45 mmol/d m² based on the geometrical cathode or anode surface area, respectively). Lower values of 15.54 ± 3.75 µmol/d (equals 11.43 mmol/d m² based on the geometrical anode surface area) for carbon laying and 14.46 ± 2.89 µmol/d (equals 28.00 mmol d m² based on the geometrical anode surface area) for DSA were obtained. DSA therefore gave the highest production rate based on the geometrical anode surface area, leading to the conclusion that the anode surface might be limiting in this case. The lowest amount of methane was produced with a carbon fabric anode. The results clearly show that the changes in methane production do not depend solely on the geometrical or specific surface areas of the anodes.

When using the graphite rod as anode, the anolyte changed its color to yellow and further to brown during the process. Also, the surface of the graphite rod roughened during the experiment. It is thus likely that the graphite rod corrodes/oxidizes when used as anode in combination with the phosphate buffer and thereby serves as a kind sacrificial anode (see pictures in the Supporting Information). Although the color change of the phosphate buffer was not observed for the other anode materials, corrosion of the carbon based electrodes might occur as well due to the oxidation potential of carbon; anode potentials between 0.69 V vs. Ag/AgCl (carbon fabric) and 1 V vs. Ag/AgCl (DSA) were calculated (see also Table 2). Although the graphite rod anode shows a very good methane production during bioelectromethanogenesis, it is not a suitable anode material because of the corrosion during the process, which limits the lifetime of the system. Consequently, activated carbon felt turned out to be the most suitable material, since the absolute methane production was the highest and no oxidation of the electrode material was observed; DSA, which offers a higher specific methane production rate based on the anode surface is limited for usage in a process in its current geometrical confirmation since the material is very space consuming at low geometrical surface areas. However, oxidation of activated carbon felt could not be neglected; it is assumed that oxidation took place with all carbon based anode materials, since the anode potentials are always similar to that during the experiments with graphite rod anodes. To use DSA, the grid structure could be altered to allow larger geometrical surface areas within the reaction volume.

**Figure 2:**
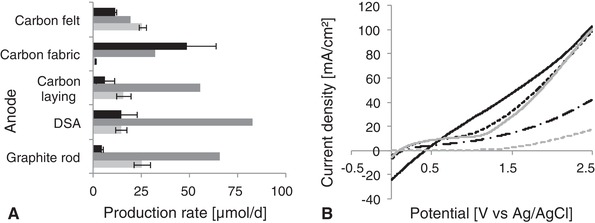
Gas production rates in H‐cells using different anodes. (A) Gas production at the cathode side in bioelectromethanogenesis; Black bar: biotic hydrogen production; dark grey bar: abiotic hydrogen production; light grey bar: biotic methane production. (B) Linear sweep voltammetry of different anodes, current density calculated based on geometrical surface area anode; solid black line: carbon fabric; dotted black line: carbon felt; dot/dash black line: graphite rod; dotted grey line: carbon laying; solid grey line: DSA

Interestingly, no direct correlation was observed between the abiotic hydrogen production in the abiotic control experiments and the methane production in the experiments with *M. maripaludris* (Figure 2A). For graphite rod, DSA and carbon laying it seemed that a high abiotic hydrogen production was responsible for a high biotic methane production and the majority of the methane is explainable by an indirect electron transfer via H_2_ (65 % in case of the graphite rod, 90 % in case of the carbon laying and 142 % in case of the DSA, hydrogen observed in biotic set‐ups not taken into account; therefore, percentages above 100 % are possible). The carbon fabric anode led to a smaller amount of abiotic hydrogen production, whereas the methane production in the biotic experiment was low, but the hydrogen production in the biological system was increased. It was already reported that *M. maripaludis* might secret hydrogenases which catalyze the hydrogen production 18; a lack of abiotically produced hydrogen might favor the secretion of hydrogenases in this case, resulting in a high biotic hydrogen production (Table 2). The methanogens might have lost the ability to produce methane due to a metabolic shift towards hydrogenases production and release. However, this effect was not confirmed when looking at the carbon felt anode and has to be stated as speculative. Although little hydrogen was produced abiotically with a carbon felt anode, the methane production was higher than for the other electrodes with a higher Coulombic efficiency of 56.1%. Only 18% of the methane produced can be explained by indirect electron transfer via abiotically produced hydrogen.

For the two materials with high methane production rates (carbon felt and graphite rod), high Coulombic efficiencies were calculated (Table 2), resulting in the conclusion that the electrical current was not the main limitation given by the anode reaction. For the two materials with medium methane production rate (carbon laying and DSA), the Coulombic efficiencies were around 25%, whereas for the carbon fabric anode, only 2% of the transferred electrons could be found in the desired product, while more than 25% were found in hydrogen. The Coulombic efficiency did not reach 100%, suggesting an alternative electron sink. Since not further organic products were detected, alterations of surface charges on the electrodes or shifting ion charges in the medium could result in lower electron flux towards the desired product. Calculating the Coulombic efficiencies for the abiotic production of hydrogen in the abiotic controls in H‐cells, it was observed that only when using DSA (100.9%) and graphite rod anode (71.5%) the electrodes were efficiently used for hydrogen production at the applied potential. For carbon laying (55.7%) and especially carbon felt (24.2%) and carbon fabric (24.1%), other electron acceptors or side reactions seemed to play a major role in the current flux. Interestingly, carbon based materials, which only differ in their structure but not in their basic material already show a high impact on the process. Our results show that the changes in the process performance cannot be explained by the geometrical or specific surface areas of the anodes. Obviously, the surface properties of the chosen anode material have a great impact on the methane production at the cathode.

### 3.2 LSV of different electrodes

In the LSV experiments, the different materials showed different current densities at applied potentials between 0 and 2.5 V vs. Ag/AgCl (Figure 2B). The current densities were calculated based on the geometrical surface area of the respective anodes. The highest current density was observed for the carbon fabric electrode, but due to the steep ground slope it seemed that a large proportion of the current results from internal electrode resistance and a capacitive behavior. Excluding the carbon fabric, the LSV revealed that for the anode potential of +1 V vs. Ag/AgCl, the carbon felt and DSA lead to the highest current density. With increasing potential up to 2.5 V vs. Ag/AgCl carbon felt and DSA show similar increasing current density curves, whereas the increase of the current density of the graphite rod curve was smaller. These results suggested that especially carbon felt and DSA led to the highest specific water splitting reaction rate, the highest specific oxygen production rate and the highest specific production of protons in the anode chamber. Since the geometrical electrode surface area of the DSA was smaller than that of the felt, it seems obvious that a larger absolute current, oxygen evolution rate and proton release rate occur in when using carbon felt anodes. In general, the higher proton release at the anode is likely to also increase the proton availability at the cathode side due to the use of a proton exchange membrane, which explains higher methane production rates. To a certain content, this is also valid for the graphite rod electrode. The increased electron flux to the cathode when using anode materials with high current densities during LSV also allowed an increase of direct and indirect electron transfer to the microorganisms in bioelectromethanogenesis. Carbon laying shows lower current densities, a proportional link to the methane production rates could not be observed. The production of abiotic hydrogen was even less predictable from the LSV. Actually, high current densities should allow higher abiotic hydrogen production rates, but the carbon fabric, which showed a high current density in LSV led to low abiotic hydrogen production rates compared to the other materials.

Based on the total surface area instead of the geometrical surface area, the current densities obtained would look different; the highest current density would then be observed for the carbon laying, followed by the carbon felt. The lowest current density based on the total surface area resulted from the use of the carbon fabric due to its high specific surface area, leading to the question why this material often works well as anode in MFC set‐ups; it seems that only minor parts of the total surface area actively participate in the reaction. However, apart from water splitting and oxygen evolution, electrode oxidation should also be considered as possible anodic reaction during LSVs, which is valid for all carbon based electrode materials.

All in all, only the internal anode resistances and partially the current density observed in the LSV seemed to correlate with the biotic methane production. A lower internal anode resistance would lead to a lower overall system resistance, resulting in lower energy losses. This could allow higher current densities in the biotic experiments at constant working potentials, and therefore higher methane production rates. Total surface area, anode mass and abiotic hydrogen production did not show a predictability of the biotic performance. A table comprising all electrode material properties and performance is given in the SI.

In general, the performance might be further increased by the use of precious metal anodes, but this would lead to increasing costs and is therefore usually not considered in bioelectrochemistry.

### 3.3 Effect of anolyte

**Figure 3:**
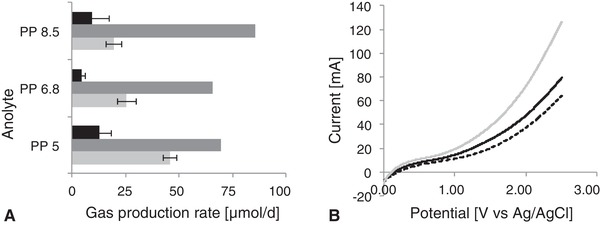
Gas production rates in H‐cells using phosphate buffer with different pH as anolyte. (A) Gas production at the cathode side in bioelectromethanogenesis; Black bar: biotic hydrogen production; dark grey bar: abiotic hydrogen production; light grey bar: biotic methane production. (B) Linear sweep voltammetry using different anolytes; solid black line: pH 6.8 dotted black line: pH 5.0; solid grey line: pH 8.5

Apart from different anode materials, different anolytes were tested, using an acidic, neutral and basic phosphate buffer. In LSV experiments, the currents observed at the potential of +1 V vs. Ag/AgCl are relatively similar, with the highest current observed at the higher (basic) pH, the lowest at the lowest (acidic) pH (Figure 3B). Against this finding, the acidification of the anolyte improved the methane production by 1.6 (Table 3). The use of a more basic phosphate buffer did not significantly alter the performance, but the pH of the anolyte measured after the chronoamperometric experiment was 6.8 in biotic and abiotic experiments when starting at pH 8.5. Protons produced at the anode seem to decrease the anode pH because the proton transport through the membrane is either slower than the proton release or limited by the proton gradient in the other direction, resulting in a neutral pH. Therefore, the methane production rate in experiments with a basic anolyte is similar to the one observed using the neutral phosphate buffer from the beginning.

**Table 3:** Performance in H‐cells using different phosphate buffers as anolyte

pH phosphate buffer	Current [mA]	Methane production rate [µmol/d]	Hydrogen production rate [µmol/d]	Coulombic efficiency to methane [%]
5	−0.89 ± 0.09	45.64 ± 3.21	12.86 ± 5.18	46
6.8	−0.56 ± 0.02	25.31 ± 4.42	4.42 ± 1.33	40.1
8.5	−0.44 ± 0.04	19.29 ± 3.86	9.32 ± 7.77	39.1

A possible explanation for the improved performance using acidic buffer at the anode is the higher proton availability, leading to an increased proton transfer to the cathode chamber, which allows an increased methane production. The larger amount of protons at the cathode could also lead to a better hydrogen production, thus providing hydrogen for an increased indirect electron transfer for the methane production. However, the hydrogen production rates, neither biotic nor abiotic differ significantly enough to finally confirm this hypothesis (Figure 3A); it might be that the increased proton flux only occurred in the biotic experiments, since the uptake of protons by the microorganisms increased the concentration gradient between anode and cathode chamber, improving the proton flux through the proton exchange membrane. Apart from protons, K^+^ and Na^+^ are likely to cross the membrane 23, especially if the proton availability due to basic pH is limited; acidification increases the selectivity of the membrane towards proton transport.

To further examine the influence of anodic pH on the process, experiments were conducted using 0.1 M NaOH and 0.1 M HCl as anolyte, respectively (Table 4). With HCl solution as the anolyte, a high hydrogen production in the abiotic (2.25 mmol/d) as well as in the biotic (0.6 mmol/d) experiments was measured, while no methane could be detected. The pH after the chronoamperometric measurement in the anode chamber was 1.87 and in the cathode chamber 2.41, instead of the initial neutral pH. This revealed that protons from the anode migrated through the proton exchange membrane, leading to an acidification of the cathode and consequently an inhibition of the cells. The pH optimum for *M. maripaludis* lies in the range between 6.8 and 7.2 21. Furthermore, the use of HCl solution as anolyte led to complete dissolving of the graphite rod anode (see picture in Supporting Information). With NaOH solution at the anode, a higher mean methane production rate compared to that observed with phosphate buffer pH 6.8 as anolyte was observed, but the deviation of this experiment was larger than in other experiments, leading to a non‐significant increase. An increase in methane production was observed using MES medium as anolyte, probably due to the higher medium conductivity (41.5 mS/cm instead of 32.7 mS/cm in the phosphate buffer pH 6.8, see Table 3). However, the use of acidic phosphate buffer resulted in a methane production similar to that observed with the MES medium, so the use of costly media with high salt contents is not required.

**Table 4:** Performance in H‐cells using different anolytes

Anolyte	Current [mA]	Methane production rate [µmol/d]	Hydrogen production rate [µmol/d]	Coulombic efficiency to methane [%]
NaOH solution	−0.6 ± 0.1	34.55 ± 18.48	24.64 ± 11.25	60.3
MES medium	−0.84 ± 0.07	33.98 ± 11.48	30.3 ± 19.1	42.5

## 4 CONCLUDING REMARKS

For the first time, different anode materials and anodes were compared regarding their impact on cathodic MES. It was shown that the anode chamber strongly influences the overall process of bioelectromethanogenesis, although the anodic reaction is always supposed to be a water splitting reaction. All in all, the anode material showed a higher impact on the process than the electrolyte; the methane production rate differs by a factor of 26.5 between the worst and the best anode material tested. Using LSV, a rough estimation can be made whether a material might be suitable or not, especially when compared to other electrode materials; a suitable material shows a high current density at the desired working potential in the LSV, combined with a low internal and contact resistance and material stability in under the respective conditions.

The influences observed are explainable, but still, the performance with different cathode materials stay relatively unpredictable from observations in abiotic experiments; for an optimization, it is not sufficient to only investigate the abiotic electrode behaviour of the anode, it shall always be tested in the biotic experiment as well. This publication showed that the optimization of the anode material and the anolyte significantly influence the cathodic MES process of bioelectromethanogenesis. However, it is not yet possible to make general statements about the effects and reasons of these improvements. For the elucidation of the underlying mechanisms further investigations are required (e.g., more electrode materials, different potentials, different electrode surfaces). Apart from that, designs with decreased system resistances (e.g., using larger membrane areas) might help to decrease the anodic overpotentials and avoid anode oxidation. In summary, our investigations show that the optimization of the anode reaction has great potential for optimizing the overall process of MES. Together with current research about the scalability and stability of the process 22, 24, this optimization possibility can be a further step on the road to industrial application.

## CONFLICT OF INTEREST

The authors have declared no conflict of interest.

## Supporting information

Supplementary Information
