# Supplementary material for: Insights in the anode chamber influences on cathodic bioelectromethanogenesis – systematic comparison of anode materials and anolytes
Source: Eng Life Sci. 2019 Sep 30;19(11):795–804. doi: 10.1002/elsc.201900126 (PMC6999415; doi:10.1002/elsc.201900126)
Supplement: Supplementary file 1 — Supplementary Information [file ELSC-19-795-s001.pdf]

Supplementary Information

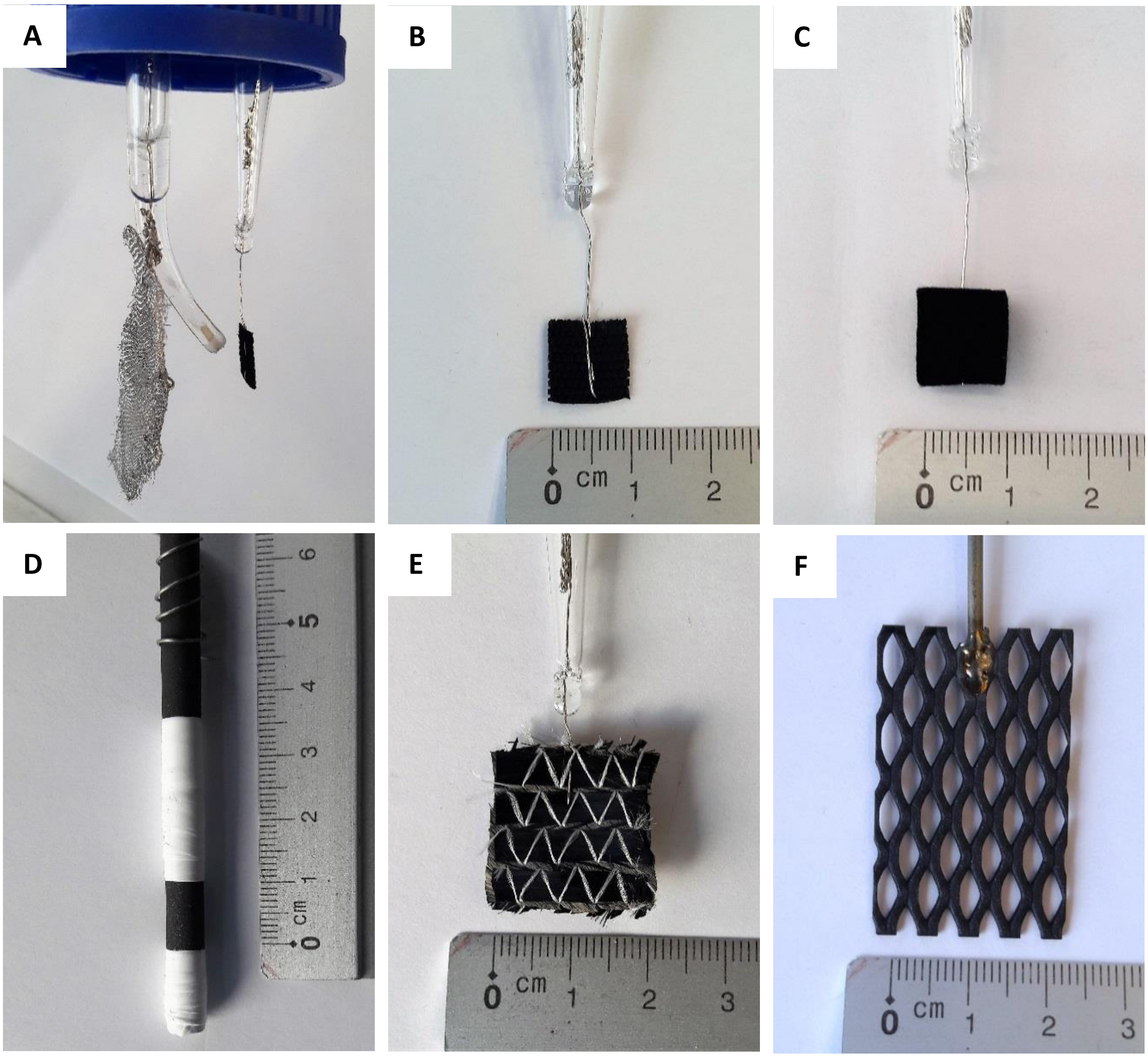

Figure 1: Series of images of the linear sweep voltammetry experiments. A: exemplary experimental set-up of CV testing. B: Carbon Fabric connected to Pt-wire. C Carbon felt connected to Pt-wire. D: Graphite rod connected to Ti-wire. E: Carbon layer connected to Pt-wire. F: Dimensionally stable anode DSA.

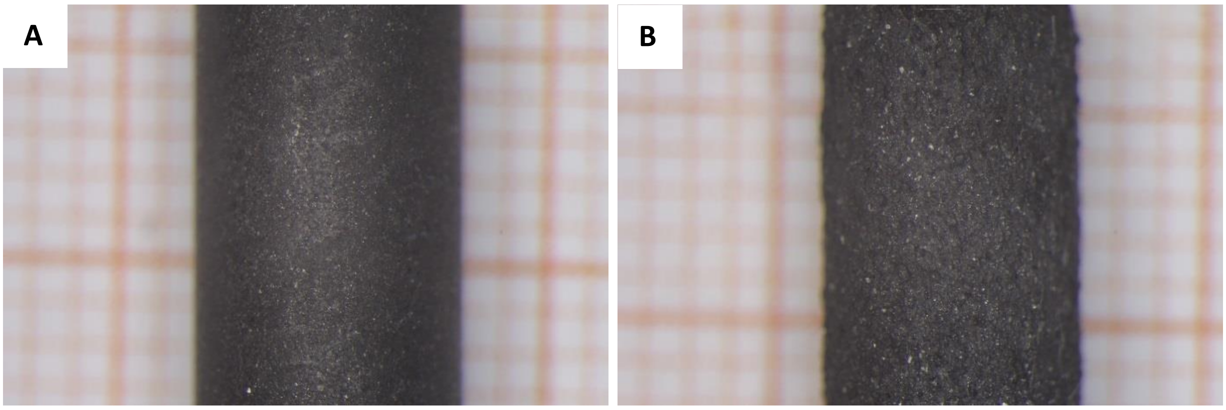

Figure 2: Photographs of graphite rods. Image A shows a graphite rod prior to the use in a synthesis experiment. Image B shows a dismounted graphite rod after use as anode in a synthesis experiment.

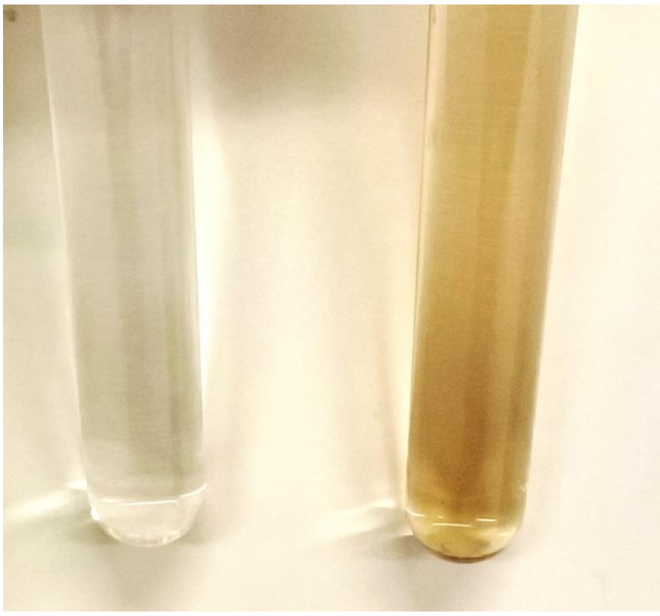

Figure 3: Electrolyte color change after corrosion of the graphite rod anode.

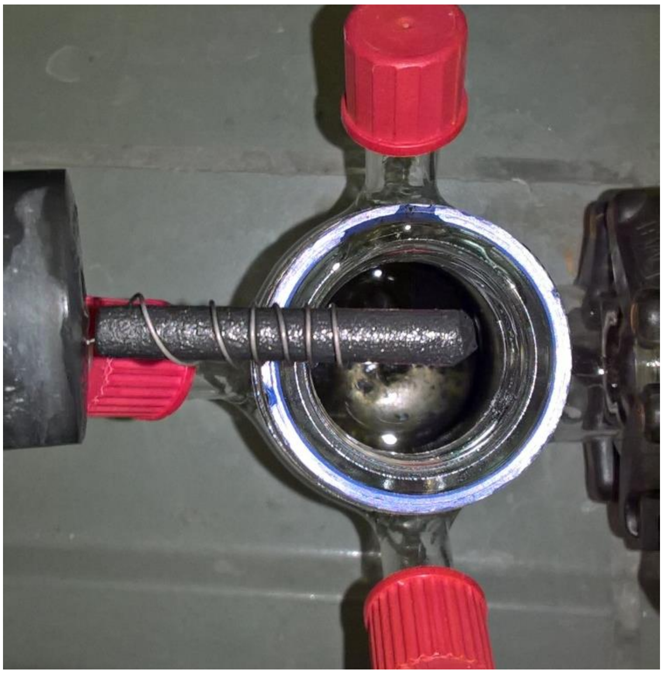

Figure 4: Corrosion of the graphite rod anode after use in 0.1 M HCl

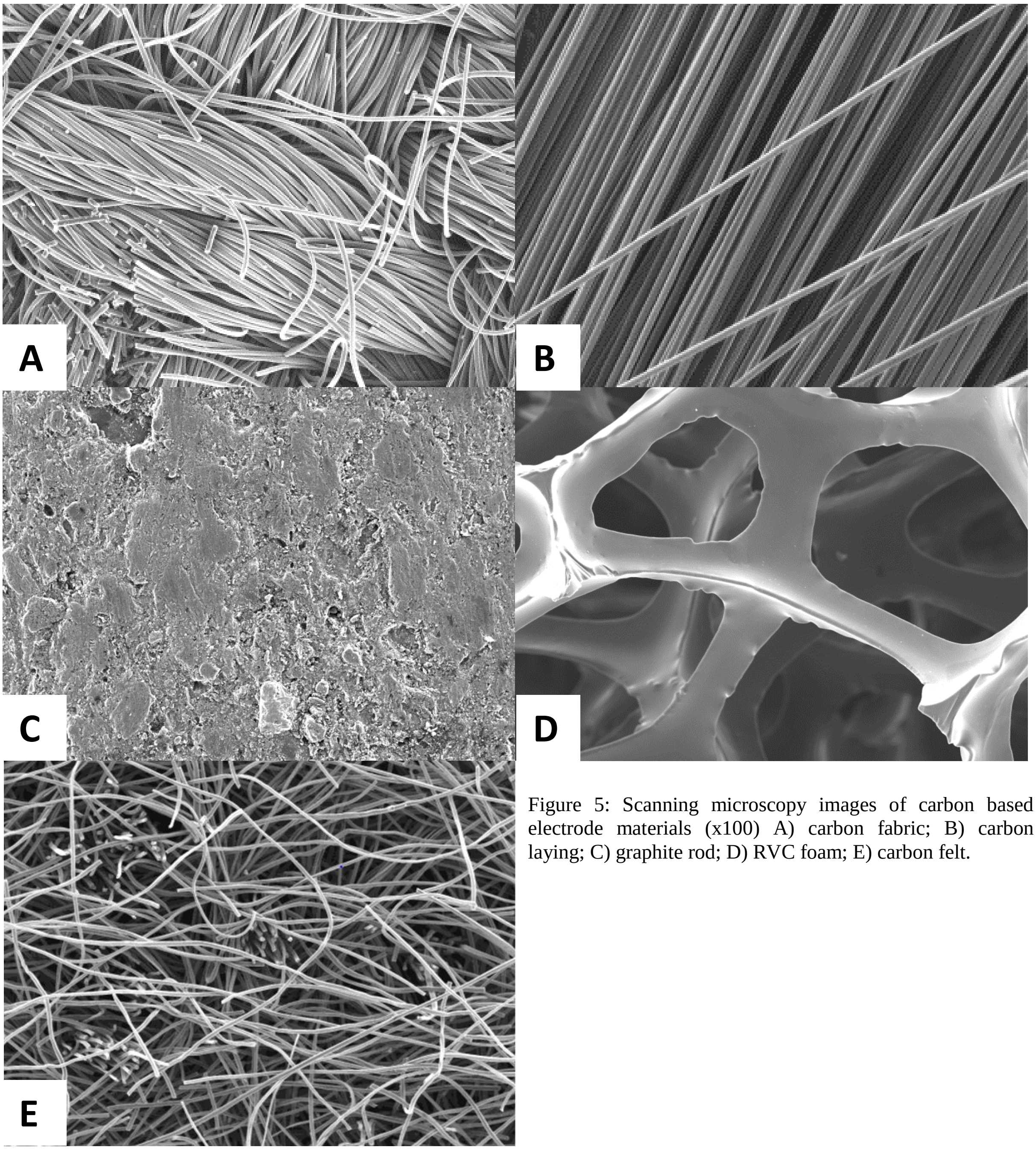

Figure 5: Scanning microscopy images of carbon based electrode materials (x100) A) carbon fabric; B) carbon laying; C) graphite rod; D) RVC foam; E) carbon felt.

Table 1: Properties and performance of different anode materials used. Green: “good” value.i.e. low value in case of resistances, large value in case of production rates, surface area and efficiencies, Yellow: “medium” value, red “bad” value

Property/Performance	Unit	Graphite rod	Carbon felt	Carbon fabric	DSA	Carbon laying
Specific electrode resistance	mΩ *m	0.05	1.41	13.98	5.7	1.63
Geometrical surface area	m²	0.00118	0.0016	0.0014	0.0005	0.0014
Specific surface area	m²/g	25.231	39.974	1635.293		0.888
Material density	kg/m³	1595.7	96.8	283.3		383
Total anode mass applied	g	3.15	0.27	0.27		0.75
Total anode surface area applied	m²	79.43	10.71	446.60		0.66
Contact resistance	Ω	0.7	3.1	109	0.5	4.4
Mean abiotic current	mA	-0.21	-0.18	-0.3	-0.18	-0.22
Mean biotic current	mA	-0.56	-0.4	-0.43	-0.48	-0.56
Mean H_2 production rate abiotic	mmol/d	0.066	0.019	0.032	0.083	0.056
Mean H_2 production rate biotic	mmol/d	0.004	0.011	0.049	0.015	0.006
Mean CH_4 production rate biotic	mmol/d	0.025	0.025	0.001	0.014	0.016
Coulombic efficiency to H_2 (abiotic)	%	71.5	24.2	24.1	100.9	55.7
Coulombic efficiency to H_2 (biotic)	%	1.8	6.2	25.3	6.9	2.4
Coulombic efficiency to CH_4	%	40.1	56.1	2.0	26.9	24.8
Mean terminal voltage abiotic	V	-1.623	-1.930	-5.518	-1.951	-1.850
Mean terminal voltage biotic	V	-1.785	-1.734	-1.594	-1.897	-1.849
